# Fulminant Cerebral Fat Embolism: Case Description and Review of the Literature

**DOI:** 10.1155/2018/7813175

**Published:** 2018-07-11

**Authors:** Giorgio Berlot, Rossana Bussani, Vennus Shafiei, Nadia Zarrillo

**Affiliations:** ^1^University of Trieste, Dept. of Anesthesia and Intensive Care, Cattinara Hospital, Strada di Fiume 447, 34149 Trieste, Italy; ^2^University of Trieste, Dept. of Pathology, Cattinara Hospital, Strada di Fiume 447, 34149 Trieste, Italy; ^3^Caserta Hospital, Dept. of Anesthesia and Intensive Care, Via Ferdinando Palasciano, 81100 Caserta, Italy

## Abstract

The release of fat and bone marrow fragments is a common occurrence following traumatic and nontraumatic events. In most cases, they go symptomless or cause only minor disturbances, but occasionally they can determine a multiorgan dysfunction whose severity ranges from mild to fatal. The authors describe the case of a patient who became deeply comatose and ultimately died after a traffic accident in which he suffered the exposed right femoral and tibial fracture in the absence of other injuries. He underwent the external fixation of the fractured bones 2 hours after the admission under general anesthesia. Three hours later, he failed to awake at the suspension of the anesthetic agents and became anisocoric; a CT scan demonstrated a diffuse cerebral edema with the herniation of the cerebellar tonsils; these abnormalities were unresponsive to the treatment and the brain death was one day later. The causes, the mechanisms, the symptoms, the prevention, and the treatment of the syndrome are reviewed and discussed.

## 1. Introduction

A number of traumatic as well nontraumatic circumstances can determine the passage of bone components such as fat and hemopoietic tissue into the bloodstream [1–3]. Independently from the triggering event, the ensuing fat and bone marrow emboli (FE and BME, respectively) can reach the systemic circulation crossing the lung capillaries, through intrapulmonary physiologic shunts or via a patent foramen ovale (PFO) [4].

In most cases, FE and BME cause no or only minor clinical consequences and only few patients develop systemic complications, a condition usually known as Fat Embolism Syndrome (FES). However, despite its widespread use, this denomination ignores the relevant role played by the BME especially in younger subjects. Whatever acronym is used, two different and not mutually exclusive mechanisms have been hypothesized in its pathogenesis: the first consists in the obstruction of the microvascular network by one or both components (mechanical hypothesis) whereas the other advocates the irritative effects exerted by the free fatty acids released by the interaction between the FE and the lung lipases on the endothelium and the subsequent activation of the inflammatory and coagulative cascades (biochemical hypothesis) [4].

The subsequent dysfunction(s) and related symptoms can occur alone or in combination and are primarily related to the end organ(s) involved. The most common clinical presentations include a noncardiogenic pulmonary edema, disturbances of the central nervous system (CNS) of variable severity, and coagulative alterations. Remarkably, despite the high number of predisposing circumstances, the occurrence of a full-blown FES is reported only occasionally [5, 6]. In the absence of a specific treatment, the early fixation of long-bone fractures and the avoidance of the intramedullary reaming represents the only measures that are able to prevent the syndrome and/or to reduce the related complications [6, 7].

Here we describe the case of a trauma patient who developed a fulminant cerebral FES approximately two hours after the trauma when he underwent urgent orthopaedic surgery under general anesthesia.

## 2. Case Description

**Figure 1:**
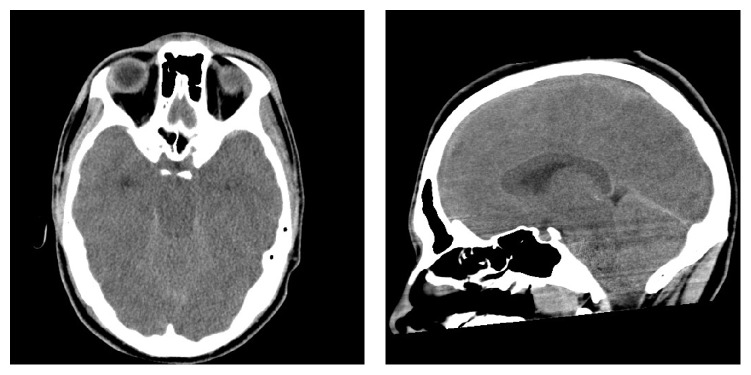
a (left): brain CT scan showing a diffuse edema with the disappearance of the gray/white matter limitation; b (right): herniation of the cerebellar tonsils.

A 17-year-old man was involved in a road accident in which he suffered the open fractures of the right femur and tibia. At the arrival to the Emergency Dept (ED), he was alert and hemodynamically stable and the Glasgow Coma Scale (GCS) was 15; the initial alignment of the fractured ends was performed in the ED with a gentle traction performed under sedation with iv. ketamine; a total body CT did not demonstrate other injuries. Approximately two hours after the admission the patient was taken to the surgical theatre for the external fixation of the fractured bones; at entering the operating room, the GCS was 8, the arterial pressure was 115/80 mm Hg, the heart rate was 115 bpm, and the arterial oxygen saturation (SPO_2_) was 85 at room air; the procedure was performed under general iv anesthesia with propofol and remifentanyl; the standard monitoring included the ECG, the noninvasive arterial pressure, the SPO_2_, and the end-tidal CO_2_ (ETCO_2_); during the intervention, the SPO_2_ rose to 100% at a FIO_2_=40% and all the other variables remained stable throughout the procedure after the 3-hour-long intervention in which the complete alignment of the bony ends was achieved; the patient was transferred to the Intensive Care Unit (ICU) still intubated and mechanically ventilated; the iv anaesthetics were gradually tapered until the complete suspension. Two hours later, the SpaO_2_ and the ETCO_2_ slightly decreased and anisocoria was observed; and an urgent CT scan of the head demonstrated a diffuse cerebral edema and the herniation of the cerebellar tonsils (Figures 1(a) and 1(b), respectively). At this time, the pupils became bilaterally mydriatic and the EEG was almost isoelectric; due to the severity of the conditions, a MR scan was considered unnecessary. On the basis of the clinical and radiologic findings repeated boluses of iv. mannitol and steroids were given in the following hours aiming to reduce the intracranial pressure. An echocardiogram demonstrated a severe right ventricular depression with an ejection fraction of 20%. On the following day, the patient was declared brain dead according to the current Italian law.

**Figure 2:**
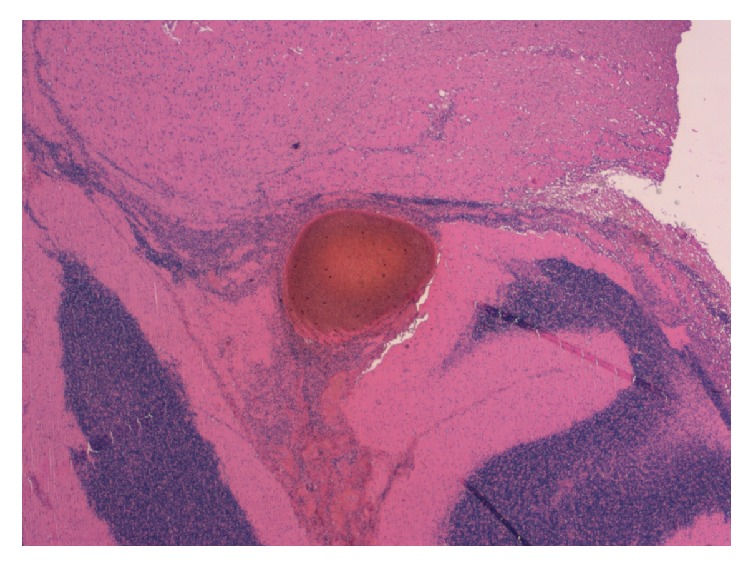
Large vascular thrombus in the cerebellum (H & E, *∗* 2.5).

**Figure 3:**
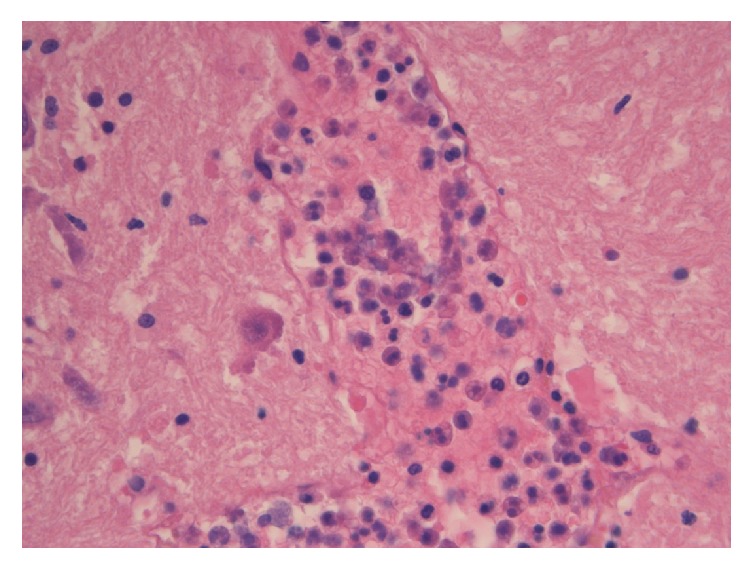
A cerebral vein completely obliterated by normally noncirculating bone marrow components (promyelocytes and myelocytes) (H & E, *∗*40).

**Figure 4:**
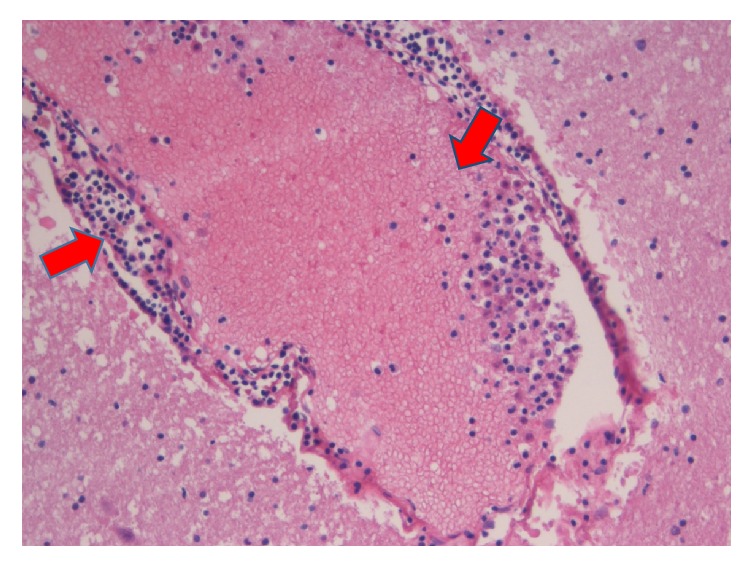
Venular occlusion by erythrocytes, promyelocytes, and myelocytes (arrows) (H & E, *∗* 20).

**Figure 5:**
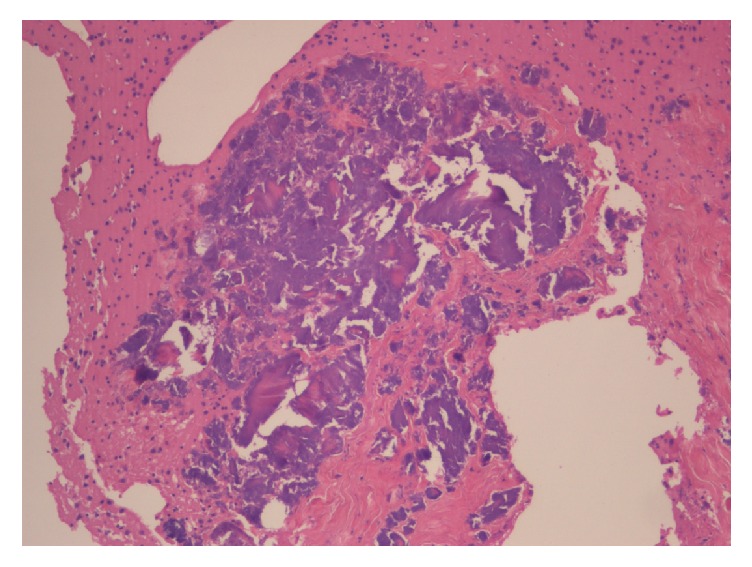
Coarse calcification in the cerebral parenchyma (H & E, *∗* 10).

At the autopsy, the cerebral microvascular network appeared diffusely plugged with BME (Figures 2–4) and ischemia-related microcalcifications were scattered throughout the brain (Figure 5); other organs were less extensively involved; no PFO was demonstrated.

## 3. Discussion

In trauma patients, the diagnosis of FES can be challenging because the symptoms (a) are not specific, ranging from mild dyspnoea to severe disturbances of the central nervous system (CNS) and possibly death; (b) appear after variable intervals of time after the injury; and, finally, (c) can overlap with those caused by the initiating event. Then, it is likely that this clinical entity goes largely undiagnosed either in adult or in pediatric patients [11–13] as reflected also by the wide variations of its incidence and time of onset reported in different studies, ranging from ≤ 1% to 35% and from ≤ 6 to > 48 hours, respectively [2, 14].

Once triggered, the outcome of patients with FES is related to different circumstances, including the amount of BME and FE released, their final location, and the severity of the end organ dysfunction. The fulminant clinical course and the poor outcome of our patient despite the immediate external fixation, which is considered the gold standard for patients at risk, deserve some remarks. [15, 16].

First, the double long-bone fracture likely determined a massive release of FE and BME. Actually, both their amount and the velocity of bloodstream invasion have been associated with the outcome: Kamenar et al. [16] observed up to 100 fat globules /mm^2^ of brain area in an autoptical study performed in a patient with a femoral shaft fracture and Cui et al. [17] demonstrated that the velocity of infusion of BM-derived mesenchymal stem-cells as well as their dose was positively associated with the appearance of cerebral ischemic lesions in a rodent model of cell-based therapy for stroke. More recently, also Jarmer et al. [18] observed a correlation between the severity of the fractures, the amount of pulmonary FE, and the outcome. As occurred in our patient, FE and BME can get the systemic circulation via pathways other than a PFO, including the lung microvascular network and the intrapulmonary physiologic shunts.

Second, it is likely that the embolic spread towards the brain initiated immediately after the trauma and continued during both the initial stabilization and the intervention due to the manipulations of the fractured bone ends; the progressive ischemic and cytotoxic damage to the lung and the brain caused by the FE and [4] likely account for the deterioration of the SPO_2_ and of the neurologic conditions observed at the arrival in the surgical theatre. Actually, it appears that both the severity and the timing of the initial CNS symptoms are related to the outcome: in a recent review, Kellogg et al. [19] reported that no or only minor mental status changes, focal deficits, or seizures at the admission were associated with a good outcome in 90,6% of patients, but this rate dropped to 57,6% in the presence of coma or abnormal posturing; other investigators demonstrated that the duration of the free interval is associated with the outcome, being worse in patients whose symptoms were present already in an earlier phase (1-8 hours) as compared with those whose neurological deterioration appeared later on [14, 19]. As far as the imaging is concerned, the CT scan is considered not sensible enough especially in the initial phases of the disease and a MR scan is warranted in order to detect the scattered (so-called “starfield pattern”) or confluent areas of cytotoxic edema caused by the plugging of the cerebral vascular network [19]. This imaging was not obtained in our patient due to the absence of any therapeutic option other than the administration of mannitol and steroids able to reverse or limit the already established severe neurologic damage.

Third, both the SPO_2_ and the ETCO_2_ did not change during the surgical procedure, possibly due to the better cardiopulmonary functional reserves of a young subject, and started to deteriorate only after the ICU admission. Actually, the lack of specific signs and symptoms directly ascribable to BME of FE makes the diagnosis elusive and a number of scoring systems have been developed to overcome these difficulties (Table 1) [4, 8, 9, 20]; however, all of them require, among other signs, a deterioration of gas exchanges in association with acute changes in CNS function; yet, we could not observe alterations of these due to the general anesthesia and mechanical ventilation.

Fourth, the presence of BME is far less common that that of FE: actually, Dettmer et al. [10] studied a group of 982 patients (age 75,7±11,9 years) who died following trauma and orthopaedic procedures and observed that (a) a BE or a FE was present in only 34 (3,4%) cases; (b) a BME was present in only 3% of them; and (c) they were mutually exclusive, the BE being primarily associated with the resuscitative manoeuvres and the FE with orthopaedic procedures and/or bone disease; this finding leads the authors to hypothesize that whereas BME had been released from the ribs broken during the CPR the FE could have been derived from the subcutaneous tissue. In our patient, the remarkable amount of blood cells precursors obstructing the brain small vessels must be ascribed to their abundance in the bone marrow of a young subject as compared to that present in elderly patients observed by other investigators [10, 11] in whom the hemopoietic tissue is largely replaced by fat [21].

## 4. Conclusions

The occurrence of a trauma-associated full-blown FES represents a potentially catastrophic clinical condition whose diagnosis requires a high index of suspicion. Its severity largely depends on the extension of trauma and on the amount of BE and BME consequently released. The only risk-reducing measure is the early fixation avoiding the medullary reaming and the treatment is only supportive.

## Figures and Tables

**Table 1:** Scoring systems used for the diagnosis of FES. CNS: central nervous system; ESR: erythrocyte sedimentation rate.

**Author**	**Features**
A.R. Gurd et al. [8] (FES= 1 major + 4 minor + fat microglobulinemia)	**Major criteria** Respiratory insufficiency, CNS involvement, petechial rash
	**Minor Criteria**
	Pyrexia, tachycardia, jaundice, oliguria/anuria, thrombocytopenia, elevated ESR, fat microglobulinemia

B.G. Shonfeld et al. [9] (FES ≥ 5 points)	Diffuse petechiae (5 points)
	Alveolar infiltrates (4 points)
	Hypoxemia (paO_2_ < 70 mm Hg)
	CNS involvement (1 point)
	Fever ≥ 38°C
	Heart rate > 120 beats/min Respiratory rate > 30 min

B.G. Lindeque et al. [10] (FES = femur fracture ± tibial fracture + 1 feature)	PaO_2_ < 60 mm Hg PaCO_2_ > 55 mmHg or pH < 7,3 Respiratory rate > 35/ min Dyspnea, anxiety, use of accessory respiratory muscles
